# Improvement of the Solubility, Permeability, and Pharmacological Activity of Decoquinate-Loaded Hydroxypropyl-*β*-Cyclodextrin–Tea Saponins Ternary ComplexA

**DOI:** 10.3390/ph18050743

**Published:** 2025-05-18

**Authors:** Wei Wei, Qihong Zhang, Weike Su

**Affiliations:** 1 Department of Pharmacology, Ningxia Medical University, Yinchuan 750004, China; 2 National Engineering Research Center for Process Development of Active Pharmaceutical Ingredients, Collaborative Innovation Center of Yangtze River Delta Region Green Pharmaceuticals, Zhejiang University of Technology, Hangzhou 310014, China; 3 Key Laboratory for Green Pharmaceutical Technologies and Related Equipment of Ministry of Education, College of Pharmaceutical Sciences, Zhejiang University of Technology, Hangzhou 310014, China

**Keywords:** decoquinate, HP-*β*-CD, tea saponin, mechanochemistry, solubility

## Abstract

**Objectives:** This study was performed to simultaneously improve the solubility, permeability, and pharmacological activity of decoquinate (DQ). **Methods:** A ternary DQ solid dispersion with hydroxypropyl-*β*-cyclodextrin (HP-*β*-CD) and tea saponin (TS) was mechanochemically prepared to enhance the efficacy of DQ. **Results:** The encapsulation efficiency of the ternary complex reached 93.51%, and the drug loading was 9.48%. The mean particle size was 90.88 ± 0.44 nm. The polydispersity index was 0.244 ± 0.004, and the zeta potential was −38.81 ± 0.75 mV. The sugar ring moiety formed multiple hydrogen bonds with the surface of HP-*β*-CD, creating favorable conditions for the development of a stable ternary complex through sophisticated molecular interactions that facilitated its assembly. In vivo studies demonstrated that the DQ/HP-*β*-CD/TS ternary complex drinking water demonstrated superior anticoccidial activity compared to pure DQ and commercial feed formulations against *Eimeria tenella*. **Conclusions:** This innovative mechanochemically synthesized ternary complex demonstrates remarkable promise for improving DQ-based formulations, as it simultaneously boosts aqueous solubility, permeability, and therapeutic efficacy. These synergistic enhancements position the compound as a strong candidate for pharmaceutical development.

## 1. Introduction

**Figure 1:**
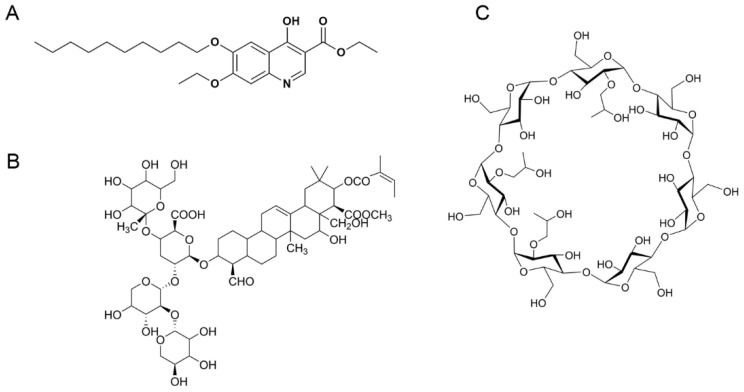
Structures of DQ (**A**), TS (**B**), and *β*-CD (**C**).

Decoquinate (DQ, Figure 1) is a poultry coccidiostat from the quinolone class [1]. This compound is approved as a feed additive to prevent intestinal coccidiosis in poultry and mammals, with years of safe use and no reported adverse effects [2]. To date, in addition to the anticoccidiosis effect, DQ has a good control effect against *Cryptosporidium parvum* [3] and *Besnoitia besnoiti* [4] infections. DQ demonstrates strong efficacy against malaria parasites in both blood and liver stages, as evidenced in rodent studies [5,6]. Although DQ has significant antiparasitic effects, DQ is extremely insoluble in water and most organic solvents. Poor oral absorption inhibits the effect of DQ. Therefore, how to improve the solubility of DQ has become a challenge.

Cyclodextrins (CDs) feature a hollow-shaped structure with a hydrophilic exterior and a hydrophobic inner cavity. The primary and secondary hydroxyl groups extend outward from the cavity’s narrower and wider edges, respectively, with the hydrogen and glycoside oxygen bridges redirected inward, making the CDs lipophilic. This special structure enables CDs to encapsulate various molecules in the cavity through noncovalent interactions, thus forming water-soluble inclusion complexes. The molecular encapsulation can well alter the physical and chemical properties of host molecules, leading to some possible benefits. For example, enhancements to the physicochemical and pharmacodynamic profiles of drug compounds may include enhanced solubility, optimized release profiles, decreased irritation, and improved stability [7]. HP-*β*-CD, a *β*-CD derivative with additional hydroxyl groups, sees the most frequent use among CD derivatives. It has favorable surface activity and internal volume enhanced drug encapsulation, as well as satisfactory inclusion ability. In particular, compared with other CDs, such as *β*-CD and methyl-*β*-CD, HP-*β*-CD exhibits relatively high water solubility and superior toxicological characteristics [8]. Therefore, HP-*β*-CD may enhance DQ’s solubility.

The inclusion molar ratio of most CDs to drugs is 1:1, forming a single molecule inclusion complex. However, DQ has a large volume and a long hydrocarbon chain, so the host–guest ratio is too low and insufficient to enhance DQ’s water solubility. Initial tests showed DQ solubility improved from 0.029 μg/mL to 1.28 μg/mL at a 1:1 DQ:HP-β-CD molar ratio. HP-*β*-CD’s high molecular weight, though, necessitates substantial formulation quantities, potentially causing host–guest inclusion imbalance, resulting in excipient consumption. High doses of CDs may cause potential toxicity and other related side effects, restricting its use as a practical and economical drug delivery option. In addition, CDs have limited encapsulation for drugs with solubility lower than 10 μg/mL and only enhance its equilibrium solubility by the encapsulation of drugs. Therefore, improving the complexation between DQ and HP-*β*-CD is highly significant. In recent years, studies have reported a possible strategy showing that the inclusion of drugs with CDs and the incorporation of a third auxiliary substance, such as a water-soluble polymer [9], into the complex can overcome the defects of the system [10,11]. In addition, by combining their respective beneficial effects, the solubility and dissolution efficiency is improved, the amount of CDs is reduced, and the effectiveness is enhanced. The external hydrophilic structure of CDs can be assembled with the third auxiliary substance through intermolecular synergy to form various aggregates [12], such as vesicles [13,14], micelles [15], layered gels, and fibrous gels, thus playing a synergistic and positive role [16].

Tea saponin (TS) is a natural small-molecule biosurfactant with an amphipathic compound isolated from the by-product of Camellia seeds [17]. This compound has attracted increasing attention and research in agriculture, food, cosmetics, and medicine. TS is environment-friendly, highly biodegradable, and biocompatible. TS exhibits a protective effect on the gastrointestinal tract without significant toxicity [18,19]. TS can be used as a stabilizer to prepare a nanosuspension for the solubilization of insoluble drugs [20]. In addition, TS possesses extensive pharmacological activities, including anti-inflammatory [21], antibacterial [21], hepatoprotective [22], and antioxidant activities [23]. TS functions as both a surface-active excipient and a pharmacologically active therapeutic agent.

This investigation aimed to concurrently enhance DQ’s solubility, permeability, and therapeutic efficacy. The current feasibility of merging DQ/HP-*β*-CD/TS complex methods to improve the solubility of DQ has not been explored intuitively. The current study was based on the combination of drugs with HP-*β*-CD complexation and hydrophilic polymer TS. The complex overcomes the poor DQ solubility and can be easily and quickly prepared without the use of a potentially harmful cosolvent, with sufficient solubility and permeability, which ensures correct dosing and therapeutic efficacy.

## 2. Results and Discussions

### 2.1. Physical Characterization of DQ/HP-β-CD/TS Ternary Complex

**Figure 2:**
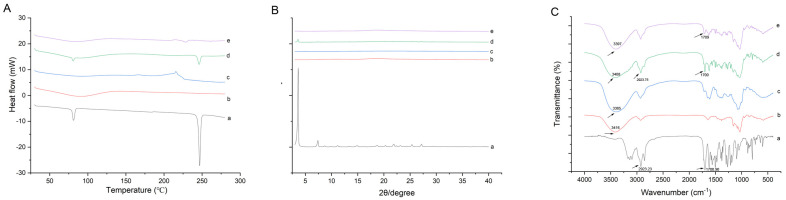
(**A**) DSC thermograms, (**B**) XRD thermograms, and (**C**) overall FT−IR thermograms of (a) pure DQ, (b) HP-*β*-CD, (c) TS, (d) PM, and the (e) DQ/HP-*β*-CD/TS ternary complex.

The results of DSC are shown in Figure 2A. During the heating process, two endothermic peaks of DQ were found at 80.9 °C and 248.1 °C. In the physical mixture (PM), DQ’s endothermic peak broadened. The characteristic crystal diffraction signals of DQ were not observed in the ternary complex, suggesting that the DQ in the ternary complex undergoes a phase transition to an amorphous form through mechanical ball milling. PXRD is the most commonly used method to analyze crystal compounds. The crystalline PXRD thermograms of the samples are shown in Figure 2B. The characteristic DQ peaks appeared at 2θ angles of 3.95°, 7.69°, 22.16°, 25.65°, and 27.52°. In the PM, DQ’s diffraction peaks diminished, and faint peaks disappeared. The DQ/HP-β-CD/TS complex showed no trace of DQ’s characteristic peak, unlike the PM diffraction pattern. The loss of drug crystallinity indicated new solid phases, suggesting ternary complex formation. The encapsulated DQ was shown to be in an amorphous form after the mechanochemical treatment by the two experimental results above. The presence of amorphous drugs is a crucial prerequisite for enhancing solubility and the dissolution rate [24,25]. This phenomenon may be advantageous in that amorphous drugs have better wettability and dispersibility; the energy required for drug dissolution in amorphous preparations is significantly less than that needed for the same amount of drugs in the crystalline state, allowing for a considerable supersaturated state to be achieved post-release.

To investigate whether new by-products were produced during the ball milling of DQ and the excipients, FT−IR spectroscopy was performed to characterize the functional groups of samples. As shown in Figure 2C, within the 300–4000 cm^−1^ spectral range, DQ had a characteristic absorption peak at 2923.23 cm^−1^, and another characteristic peak was C=O at 1700.90 cm^−1^. The FT−IR spectrum of the PM showed no additional peak shifts, indicating no chemical interaction between DQ and the excipients. In the ternary complex, a shift to 3397 cm^−1^ was observed; conversely, the associated PM presented peaks at 3408 cm^−1^. In addition, the intensity of the C=O peak for DQ shifted from 1700.90 cm^−1^ to 1709 cm^−1^ in the ternary complex, which may be due to the hydrogen bond between the DQ and excipients during the mechanical treatment. This suggests that host–guest interactions occurred, leading to new solid phases in the ternary complex versus PM.

### 2.2. Morphological Analysis

**Figure 3:**
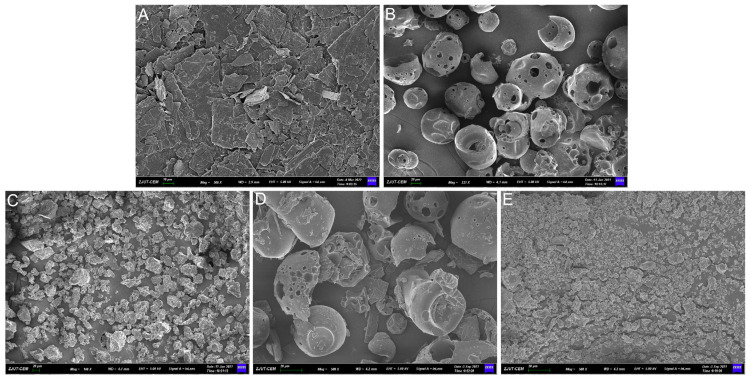
Scanning electron micrographs of (**A**) pure DQ, (**B**) HP-*β*-CD, (**C**) TS, (**D**) PM, and (**E**) DQ/HP-*β*-CD/TS ternary complex (molar ratio of 1/1/1 and milling time of 60 min).

The scanning electron micrograph in Figure 3 shows that DQ, HP-*β*-CD, and TS had crystal morphology. However, the sample crystallinity was destroyed, and polydisperse particles formed with irregular shapes under the activation of mechanochemical milling to form a solid dispersion system. The treated drug particles showed a significantly smaller diameter than untreated DQ. Decreasing the drug particle dimensions and enhancing the surface area facilitated subsequent drug dissolution processes.

### 2.3. Properties of DQ/HP-β-CD/TS Ternary Complex in Water Solution

**Figure 4:**
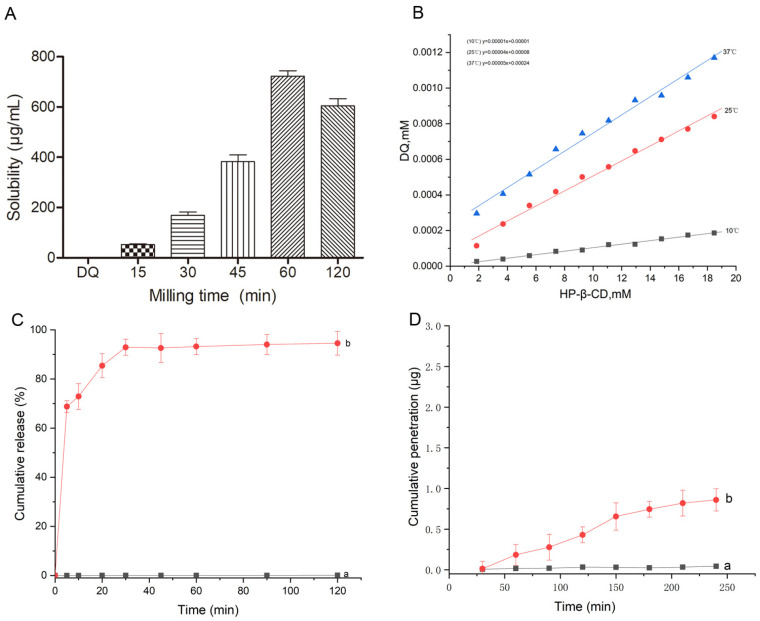
(**A**) Solubility of the pure DQ and DQ/HP-*β*-CD/TS ternary complex prepared under different milling times; (**B**) phase solubility diagrams at different temperatures; (**C**) DQ dissolution profile of pure DQ and DQ/HP-*β*-CD/TS ternary complex in water at 37 °C; (**D**) cumulative penetrations of (a) pure DQ and (b) DQ/HP-*β*-CD/TS ternary complex.

The optimum solubility of the DQ/HP-*β*-CD/TS ternary complex reached 722 μg/mL, higher than that of DQ alone, as shown in Figure 4A (0.029 μg/mL). The linear increase in DQ solubility suggests that the solubility phase analysis indicated A_L_ type (Figure 4B). The slopes were less than 1, indicating the formation of a 1:1 ternary system. For DQ, roughly 0.08% dissolved following 120 min in water, during which 94.58% DQ dissolved in 120 min the in ternary complex in Figure 4C. This pattern suggests a rapid dissolution of the complex.

The DQ’s and complex’s gut permeabilities were assessed using the PAMPA method. Compared with DQ, the DQ/HP-*β*-CD/TS ternary complex had better membrane permeability (Figure 4D). The total permeation amount attained was 0.86 μg by 240 min, and the complex effectively improved the amount of DQ passing through the intestinal tract membrane. This phenomenon could be attributed to several reasons, such as the translation of the DQ crystallinity in the complex and increased particle wettability, solubility, and drug release behavior with HP-*β*-CD and TS.

### 2.4. Characterization of Micelles

**Figure 5:**
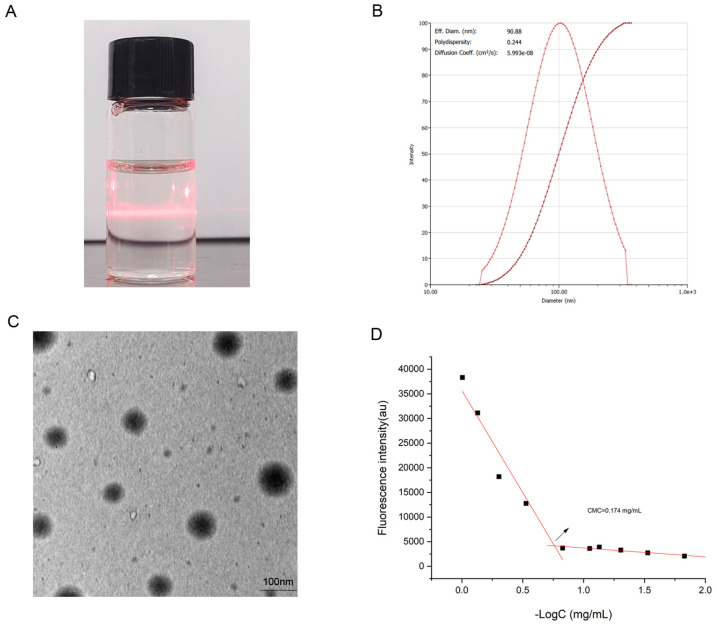
(**A**,**B**) Dynamic light scattering size measurement of the DQ/HP-*β*-CD/TS ternary complex; (**C**) transmission electron micrographs of the DQ/HP-*β*-CD/TS ternary complex; and (**D**) CMC value of the DQ/HP-*β*-CD/TS ternary complex. The arrow represents CMC.

When a laser passes through a colloidal solution, a bright “light path” appears in the vertical direction of the light due to the scattering of the light by the colloidal particles. As shown in Figure 5A, it indicates that the DQ/HP-*β*-CD/TS complex is a colloid system of a certain size in an aqueous solution. Measured via analyzer, the particle diameter registered at 90.88 ± 0.44 nm, and PDI was 0.244 ± 0.004 (Figure 5B). Figure 5C illustrates the TEM findings of the spherical complex with homogenous particle sizing, averaging ~90 nm. The CMC of the DQ/HP-*β*-CD/TS ternary complex was 0.174 mg/mL (Figure 5D), indicating that micelles could be formed at a low concentration and were extremely stable in the aqueous solution. In addition, the **ζ** potential was −38.81 ± 0.75 mV of the DQ/HP-*β*-CD/TS ternary complex. The calculated EE% and LC% were 93.15% and 9.48%, respectively (Table 1). Given the amphiphilic nature of TS, DQ could be encapsulated within the hydrophobic core of HP-*β*-CD. Furthermore, TS likely facilitates this process by engaging with DQ through hydrophobic forces while simultaneously forming hydrogen bonds with HP-*β*-CD, ultimately leading to the spontaneous assembly of micelles in an aqueous environment.

### 2.5. DQ/Biopolymers Association by ^1^H NMR Spectroscopy

**Figure 6:**
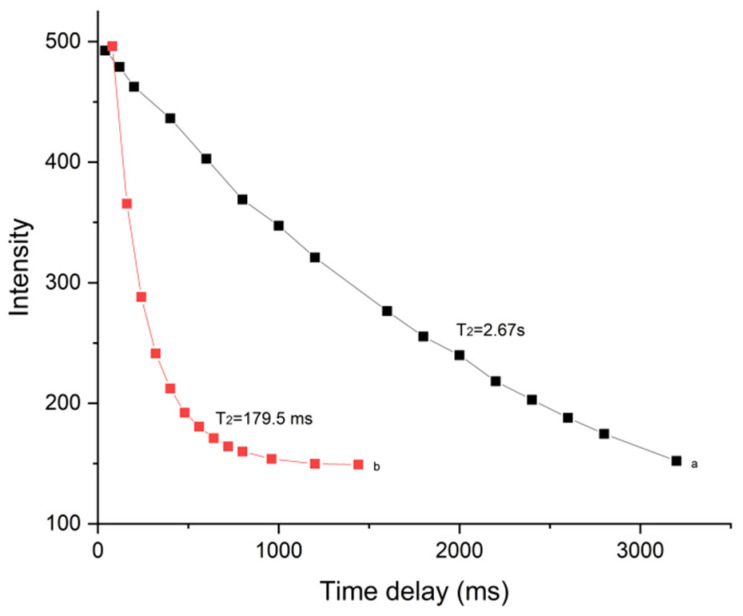
Dependence of NMR signal decay and T_2_ relaxation times of the DQ protons in water: (a) pure DQ and (b) DQ/HP-*β*-CD/TS ternary complex.

The ^1^H NMR relaxation technique was used to prove that the drug and biopolymers bound in the water solutions [26]. The T_2_ value can reflect the change in molecular environment or state. Altered T_2_ values typically indicate intricate structural formation [27]. The transverse relaxation time T_2_ results of DQ and the complex are shown in Figure 6, which also shows the T_2_ of DQ extending from 2.67 s to 179.5 ms with HP-*β*-CD and TS. This outcome offers proof of intermolecular forces and the complex formation within the system. The finding is similar to those used to increase insoluble drugs [28]. All the kinetics in the relaxation experiments showed mono-exponential time-dependence, which suggests fast guest molecule exchange between the complex and solvent.

### 2.6. Stability Study

**Figure 7:**
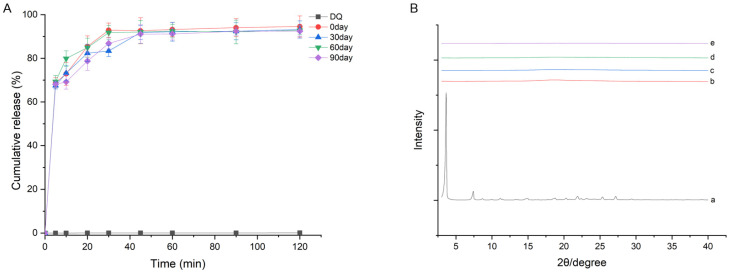
Stability test for (**A**) cumulative release at +40 °C and 75% humidity for 90 days and (**B**) XRD curves of (a) pure DQ, (b) HP-*β*-CD, (c) TS, (d) PM, and the (e) DQ/HP-*β*-CD/TS ternary complex.

The results show no crystal transformation, and the cumulative release and DQ content in the complex remained largely unchanged with the extension of storage time (Figure 7 and Table 2). Thus, the ternary complex was relatively stable in solid dispersion.

### 2.7. Molecular Docking Study

**Figure 8:**
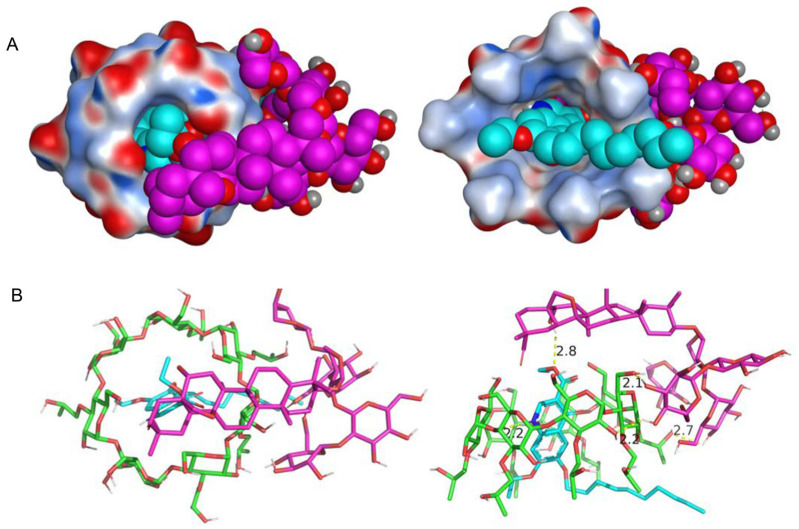
Electrostatic surface (**A**) and binding mode (**B**) of DQ with HP-*β*-CD and TS.

The docking results for the target with compounds show that DQ had good binding with HP-*β*-CD and a high matching degree (Figure 8). DQ was inserted into the HP-*β*-CD cavity, formed a good fit with the cavity, and formed a hydrogen bond interaction with the HP-*β*-CD cavity. The short hydrogen bond distance and strong binding ability can effectively improve the stability of DQ in the cavity. The hydrophobic part of the TS compound covered the top of the small rim of HP-*β*-CD, and the hydroxyl part of the TS compound formed a hydrogen bond interaction with DQ (the hydrogen bond distance was 2.8 Å), which played an important role in stabilizing the complex. The hydrophilic sugar ring was bound to the surface of HP-*β*-CD and could also form a number of strong hydrogen bond interactions with the surface of HP-*β*-CD, which can promote TS to firmly adhere to the surface of HP-*β*-CD to form a stable complex. Molecular evidence may also suggest the ternary complex enhances DQ solubility in water.

### 2.8. DQ/HP-β-CD/TS Ternary Complex Inhibited the Development of Coccidiosis

Clinical signs of *Eimeria tenella*-induced coccidiosis were promptly noticeable in the infected group: listlessness, hemorrhagic feces, and weight loss. DQ and commercial treatment are inadequate to control the clinical symptoms of coccidiosis except survival, and this phenomenon may be caused by the manner of administration. Uneven administration of the mixture hampers the corresponding anti-coccidiosis activity (Table 3). Treatment with the DQ/HP-*β*-CD/TS ternary complex abrogated the detrimental effects of coccidian infection. This was evidenced by a notable decrease in coccidian oocyst counts, a reduced lesion score, and an increased relative weight gain, while ACI was notably higher. This suggests that the combination with HP-*β*-CD and TS might enhance the effectiveness of DQ against coccidiosis in broiler chickens.

**Figure 9:**
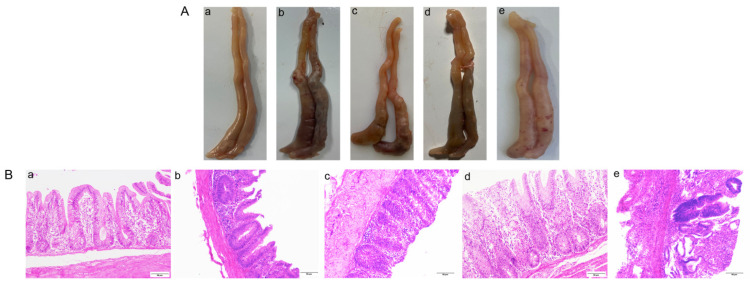
(**A**) Example images of the cecum and (**B**) HE staining results of the cecal tissue sections in each group (200×). a–e represent the control group, EI group, DQ group, DQ/HP-*β*-CD/TS ternary complex group, and DQ commercial group, respectively.

The effect of the DQ/HP-*β*-CD/TS ternary complex against cecum damage was further determined by HE staining (Figure 9B). Compared with the control group, the cecal villi in the *Eimeria tenella*-infection (EI) group were seriously damaged, while the villi in the DQ and commercial treatment groups were damaged. However, the effect was barely satisfactory. In the complex treatment group, the cecal tissue and villus structure were basically normal, and no obvious damage of the intestinal mucosa was observed. The results again illustrate that the DQ/HP-*β*-CD/TS ternary complex against coccidial infection induced cecum pathological changes.

The bioavailability of the DQ complex prepared in this study has been significantly improved compared with the active pharmaceutical ingredient. The increase in the bioavailability of decanoquine is attributed to multiple factors: (1) the prepared DQ solubilization system exists in an amorphous state in the carrier. Therefore, compared with the active pharmaceutical ingredient, DQ, with a crystalline structure, this form has a positive impact on the solubilization of DQ, since studies in the pharmaceutical industry demonstrate enhanced solubility and bioavailability in amorphous versus crystalline formulations. (2) The DQ/HP-*β*-CD/TS complex prepared by mechanical and chemical means, due to the reduction in drug particle size, demonstrates enhanced wetting and spreading properties. The porosity of the particles increases, enabling the drug to be rapidly and uniformly distributed in the carrier, increasing DQ’s solubility and dissolution rate, thereby promoting intestinal penetration and absorption. (3) For cyclodextrin, due to the hydrophobic cavity of HP-*β*-CD, it can form inclusion complexes with drugs. Although the inclusion effect on DQ is not obvious, under the action of the water-soluble polymer TS, the hydrophilic and hydrophobic interactions on HP-*β*-CD particle surfaces reach equilibrium, promoting the dispersion and distribution of DQ in the HP-*β*-CD matrix. A large number of studies have confirmed that CD can dissolve drugs through aqueous outer membranes and transport them to lipophilic membranes. Increasing the drug concentration on lipophilic membranes, that is, increasing the drug concentration through unstirred water layers, can enhance drug delivery through biofilms, thereby increasing the drug dissolution rate and improving the intestinal permeability and bioavailability of drugs. As a derivative of CD, HP-*β*-CD can also increase the permeability of DQ esters in the intestinal mucosa, improving the solubility, intestinal permeability, and bioavailability of DQ.

Of course, there are still limitations when extrapolating the research results to other species. We assessed DQ’s impact solely on E. tenella in yellow broilers. Subsequently, we will evaluate the inhibitory effect of the complex on different species and different coccidia species. Furthermore, in the subsequent research, we will monitor the temperature changes during the grinding process to make the research more comprehensive.

## 3. Materials and Methods

### 3.1. Materials

DQ (≥98.0% purity, Lot 20200810) was sourced from Wuhan Greatwall Chemical Co., Ltd. (Wuhan, China). The TS (≥98.0% purity, S9961) was obtained from Solarbio (Beijing, China), and the HP-*β*-CD, with a purity of approximately 98.0% (degree of substitution = 4.7, Lot 030168), was sourced from Shandong Zhiyuan Biotechnology Co., Ltd. (Binzhou, China). Sodium carboxymethyl cellulose (CMC-Na, product code C0603) was supplied by TCI Co., Ltd., in Shanghai, China. All the chemicals used in this study were of analytical grade quality.

### 3.2. Animals

Three 14-day-old SPF yellow broilers (100–110 g) were obtained from the Zhejiang Academy of Agricultural Sciences. The animal studies adhered to established lab animal welfare protocols.

The animals were kept in conditions with temperatures hovering between 30 and 37 °C and humidity levels of 50–60%. They had unlimited access to both fresh water, which had been boiled and cooled, and feed specifically formulated without any anticoccidial additives. Before being given to the animals, the forage was baked in a 70 °C oven for a minimum of two hours. *Eimeria tenella* was isolated from Beijing and donated by the Institute of Animal Science and Veterinary Medicine, Zhejiang Academy of Agricultural Sciences. The parasite was sensitive to current anticoccidiasis drugs. The *E.* tenella sporocysts were inoculated into 14-day-old yellow broilers. *E.* tenella sporocysts in the feces samples from the 7th to the 10th day after inoculation were collected by the saturated brine floating method [29]. The collected sporocysts were resuspended in a 2.5% potassium dichromate solution. The *E.* tenella sporocysts were sporulated at 200 rpm/min for 2 days in a temperature-controlled shaker at 28 °C and then collected and stored at 4 °C. The broilers received oral doses of sporulated oocysts, initiating infection (each chicken received 50,000 sported *E.* tenella oocysts) into the crop.

The experimental procedures received full ethical approval from the Institutional Animal Care Committee at Zhejiang Academy of Agricultural Sciences (ethics protocol No. 2021ZAASLA46, approved on 26 March 2021), following the established guidelines outlined in the National Institutes of Health’s manual on laboratory animal welfare standards.

### 3.3. DQ Ternary Complex Preparation

A Retsch PM-400 planetary mill (Haan, Germany) facilitated mechanochemical synthesis [30].

The treatment parameters were as indicated: mass of a sample, 2.2 g; drum capacity, 50 mL; and diameter and load of the grinding media (steel balls), 6.0 mm and 75 g, respectively, with a milling speed of 300 rpm. The milling times were 15, 30, 45, 60, and 120 min. DQ/HP-*β*-CD/TS was mixed at a 1:1:1 molar ratio and kept in amber vials.

### 3.4. HPLC Analysis of DQ

The analysis was performed using a ZORBAX SB-C18 column (4.6 mm × 250 mm, 5 μm; Agilent Technologies, Palo Alto, CA, USA). The method utilized a methanol–water mobile phase (90:10 *v*/*v*), with detection at 265 nm. The chromatographic conditions included a column temperature of 30 °C, a flow rate of 1.0 mL/min, and a 10 μL injection volume.

### 3.5. Content Test for DQ

The DQ levels in the sample were quantified using HPLC. We dissolved each sample in a one-to-one mix of chloroform and methanol (volume for volume). These solutions were then run through a 0.22 μm filter membrane, and a ZORBAX-SB-C18 column (4.6 mm × 250 mm, 5 μm, Agilent Technologies, Palo Alto, CA, USA) was used for separation, with a mobile phase consisting of 90% methanol and 10% aqueous solution. The flow rate was set at mL/min, and 10 μL of the solution was injected into each sample. Detection was carried out at a wavelength of 265 nm, keeping the column at a steady 30 °C.

### 3.6. Fourier Transform Infrared (FTIR)

The infrared absorption spectra were obtained using a Thermo Scientific NICOLET IS50 FT-IR spectrometer, covering the spectral range from 400 to 4000 cm^−1^. For analysis, each sample was homogenized with KBr at a 1:100 mass ratio, compressed into pellets, and subsequently subjected to spectroscopic measurement.

### 3.7. Differential Scanning Calorimetry (DSC)

The DSC thermograms were recorded using a differential scanning calorimeter (SERIES2000, Mettler Toledo Co., Ltd., Columbus, OH, USA). Under N_2_, ~5.0 mg samples underwent heating within a perforated aluminum container, progressing from 30 °C to 280 °C at 10 °C/min [31].

### 3.8. Power X-Ray Diffraction (PXRD)

Diffraction patterns were detected using the Bruker D2 PHASER diffractometer (D/max-Ultima IV, Rigaku Co., Ltd., Tokyo, Japan) at ambient temperature. The samples were irradiated with monochromatized Cu Kα radiation and analyzed in the 2θ range of 3–40°, with a scanning speed of 4° (2θ)/min. The pattern was recorded with a tube voltage of 30 kV and a tube current of 10 mA [32].

### 3.9. Morphological Characterization

#### 3.9.1. Scanning Electron Microscopy (SEM)

A scanning electron microscope (JEOL, Tokyo Co., Ltd., Tokyo, Japan) was used to acquire microphotographs on the surface morphologies. A small amount of the sample was placed in the sample cell coated with double-sided tape, the excess sample was blown off with an ear bulb, and then gold was sprayed under vacuum conditions to increase the conductivity of the sample. The operating voltage of the instrument was 15 KV.

#### 3.9.2. Transmission Electron Microscopy (TEM)

Aqueous solutions (~1 mg/mL) were created by dissolving the samples in purified H₂O. Ultrasonic treatment was carried out for 30 min to ensure uniform dispersion of the samples. A few droplets of the specimen were carefully placed onto a copper mesh grid (200 mesh) coated with carbon film and left to air-dry. The samples’ morphology was then examined using a Hitachi HT7700 transmission electron microscope (Hitachi Co., Ltd., Tokyo, Japan), operating at an acceleration voltage of 120 kV for detailed imaging.

### 3.10. Critical Micelle Concentration (CMC)

Nile red was employed as a glow-in-the-dark probe to delve into the critical micellar concentration (CMC) of our sample in a water-based environment, as noted in reference [33]. The sample, precisely measured, underwent sonication in distilled water for upwards of 30 min, yielding a uniform solution. A quantitative amount of Nile red was dissolved in acetone and placed in brown glass vials. The acetone was evaporated at room temperature. Then, different concentrations of DQ/HP-*β*-CD/TS solutions were added to the vials and shaken for 12 h at 37 °C. The emission intensity of each sample concentration was recorded at 620 nm using a spectrofluorometer (Spark, Tecan Co., Ltd., Männedorf, Switzerland), with the excitation set to 579 nm. The CMC corresponds to the inflection point observed in the plotted data.

### 3.11. Dissolution Assay

The dissolution characteristics were assessed with a dissolution testing device (RC-6, Tianjin Jingtuo Instrument Technology Co., Ltd., Tianjin, China). Each sample (50 mg) was introduced to 900 mL purified water at 37 °C ± 0.5 °C, mixing at 100 rpm for dissolution. Then, 5 mL aliquots were collected from each container at 5, 10, 15, 30, 45, 60, 90, and 120 min, then filtered using regenerated cellulose syringe filters. The sample aliquot concentration was assayed via HPLC.

### 3.12. Phase-Solubility Assay

The Higuchi–Connors technique [34], with appropriate modifications, was employed for phase solubility investigations. Excess DQ was added to the aqueous solutions containing various concentrations of HP-*β*-CD (0–20 mM). The mixtures were shaken in a thermostatic oscillator (Julabo-SW22, Julabo Co., Ltd., Seelbach, Germany) at 10 °C, 25 °C, and 37 °C at 200 rpm for 72 h to achieve equilibrium and then filtered with a 0.45 μm cellulose acetate filter membrane. HPLC measured the total DQ concentrations in the filtrates.

### 3.13. Particle Size, Zeta Potential, and Related Properties

#### 3.13.1. Particle Characterization and ζ Potential

The DQ ternary complex’s mean particle size and **ζ** potential were measured using a Nano ZS90 laser particle analyzer (Malvern Co., Ltd., Westborough, MA, USA). Aqueous solutions (~1 mg/mL) of the samples were prepared using filtered, distilled water and sonicated for 30 min. Each sample underwent three measurements at 25 °C.

#### 3.13.2. Solubility Determination

An excess amount of the sample was introduced into purified water and shaken over a 24 h to achieve equilibrium at 200 rpm in a thermostatic oscillator (Julabo-SW22, Julabo Co., Ltd., Seelbach, Germany) at 37 °C. Subsequently, the mixture was strained via a 0.45 µm filter membrane, and the DQ levels in the resulting filtrate were assessed by HPLC.

### 3.14. Drug Encapsulation Efficiency (EE) and Loading Capacity (LC)

The complex solution of DQ/HP-β-CD/TS was subjected to centrifugation at 10,000 rpm for 10 min at room temperature. The supernatant was filtered through a 0.45 μm filter membrane and was analyzed using HPLC for the DQ peak area. The drug EE% and LC% were calculated as follows:

EE% = (total mass of DQ − mass of free DQ)/Total mass of DQ × 100

LC% = Amount of DQ encapsulation in complexes/Mass of HP-*β*-CD and TS × 100

### 3.15. Parallel Artificial Membrane Permeability (PAMPA)

The PAMPA simulates drugs’ passive intestinal uptake [35]. Drug transfer efficiency from the donor to the acceptor compartment was measured using 12-well Transwell plates (Corning polyester membrane). Briefly, the dioleyl phosphatidylcholine (DOPC) solution (dissolved in hexadecane to prepare a 2% DOPC solution) was mixed with hexane in a volume ratio of 5:95 to simulate the intestinal membrane, and the chamber was ventilated overnight to ensure complete evaporation of the hexane. Recipient plates received 1.0 mL of distilled water. Then, 0.2 mL of pure DQ or DQ/HP-*β*-CD/TS ternary complex solutions were incubated in an orbital shaker at 37 °C under a shaking speed of 200 rpm for 4 h. Subsequently, 0.2 mL aliquots were removed from the receiving chamber at specific intervals and replaced with an equal volume of purified water. Extraction from the receptor plate and HPLC analysis followed.

### 3.16. ^1^H-NMR Relaxation in Solution

The NMR spectrum of pure DQ and mechanochemical activation of the complex was recorded by a Bruker NMR spectrometer in D_2_O or CD_3_OD solution (Aldrich, St. Louis, MO, USA, 99.8%; the volume was no less than 450 μL). The T_2_ was determined by the standard Carr–Purcell–Meiboom–Gill sequence of the Avance (Bruker Avance III 400 MHz Spectrometer) of the Bruker pulse sequence library: P_1_(90°)–(τ–P_2_(180°)–τ × 2–P_2_(180°)–τ)*n*–registration, where τ = 2 ms is the fixed time delay, and *n* varied from 1 to 3200.

### 3.17. Stability

To investigate the stability of the DQ/HP-*β*-CD/TS ternary complexes prepared using a mechanochemical method, the mechanically treated products of DQ were stored at 40 °C and 75% humidity for 3 months. The cumulative release, DQ content (HPLC analysis), and PXRD (D/max-Ultima IV, Rigaku Co., Ltd., Tokyo, Japan) were analyzed at 0, 1, 2, and 3 months, respectively.

### 3.18. Molecular Modeling

Molecular docking explored the potential formation pathways for the three-component complex. The Gaussian16 PM6 semi-empirical algorithm was used to optimize the structure without any constraints to obtain the optimal HP-*β*-CD geometry. DQ and TS were from the PubChem database. The energy of the downloaded compounds was minimized by Chem3D and converted into MOL2 format. The grid box dimensions (x = −3.198, y = −1.759, z = −1.296), defining the binding pocket location and spatial extent for small molecule–target interaction, were configured as 40 × 40 × 40. Pymol2.1 was used compound interaction visualization. Molecular docking was computed using a Lamarckian genetic algorithm: a maximum of 25 million energy evaluations for a population of 150 participants. Each evaluation was capped at a value of 2000, and the crossover probability stood at a substantial 0.8. The mutation rate was 0.02, and the independent docking operation was 50 times. The mutation chance was a modest 0.02, and a total of 50 independent docking trials were conducted.

### 3.19. Clinical Symptoms and Evaluation of Anticoccidial Efficiency

Post-inoculation with oocysts, the broilers were under close observation for clinical signs, including behavioral characteristics, mental state, coat color, food intake, water intake, bloody stool excretion, and death, until the conclusion of the experiment. If any animals died during the experiment, an immediate autopsy was conducted to observe the tissue lesions. On day 9 after inoculation, the relative weight gain (RWG) and survival of the broilers were measured. Freshly discharged feces were collected 7–9 days after inoculation. Subsequently, oocyst counts/gram (OPG) were determined using the McMaster technique [36]. The cecum lesion score (CLS) was determined using a five-point score, assessed via cecal wall thickness/atrophy and hematic presence [37]. The efficiency of the complex was also evaluated using the anticoccidial index (ACI).

Relative weight gain (%) = (infected group average weight gain/uninfected control group average weight gain) × 100%.

The CLS encompassed hemorrhages, cecal wall thickness, and mucus secretion, graded on a scale of 0–4 for intensity [38] (Table 4). The oocyst value was calculated based on the ratio of the number of oocysts discharged in each group to the number discharged in the infection non-administration group (the oocyst ratio). The oocyst ratio was 75–100%, with an oocyst value of 40; a ratio of 50–75% corresponded to an oocyst value of 20. A ratio of 25–50% had an oocyst value of 10, and a ratio of 1–25% corresponded to an oocyst value of 5. Finally, a ratio of oocysts to follicles less than 1% was assigned an oocyst value of 0.

### 3.20. Intestinal Histopathological Analysis

Each cecum tissue sample was washed and fixed with 10% paraformaldehyde. The ceca underwent dehydration through an alcohol series, were cleared in xylene, and were embedded within paraffin before being sliced into 5 μm sections using a microtome (Leica, Wetzlar, Germany). Intestinal sections were deparaffinized and hydrated for HE staining. The cecal tissue’s histopathology was examined using light microscopy (200×), and images were captured.

### 3.21. Statistical Analysis

The experiments were conducted in triplicate, with the outcomes reported as mean ± standard error of the mean. The statistical analyses were performed using SPSS 21.0 software (Chicago, IL, USA). The outcomes underwent one-way ANOVA analysis, followed by Dunett’s test for statistical assessment. The significance threshold was set at *p* < 0.05.

## 4. Conclusions

This research reveals that the DQ/HP-*β*-CD/TS ternary complex spontaneously forms micelles in an aqueous solution, achieving a high drug EE% of 93.51%. The ternary complex exhibited excellent solubility and permeability in vitro. Molecular docking studies supported the ternary complexation hypothesis. The ternary complex showed excellent anticoccidial efficacy against *Eimeria tenella* infection. Consequently, it is posited that the collaboration between DQ, HP-*β*-CD, and TS may significantly alter DQ’s physicochemical and biological characteristics, such as the improvement of DQ solubility, dissolution, permeability, and anticoccidial efficiency, potentially harboring significant pharmaceutical prospects.

## Figures and Tables

**Table 1:** The ζ potential, average size, PDI, EE, LC, and aqueous solubility of DQ/HP-*β*-CD/TS ternary complex (n = 3).

Sample	Solubility (μg/mL)	ζ Potential (mV)	Size (nm)	PDI	EE (%)	LC (%)
DQ ternary complex	722.41	−38.81 ± 0.75	90.88 ± 0.44	0.244 ± 0.004	93.51	9.48

**Table 2:** The content of the DQ/HP-*β*-CD/TS ternary complex in the stability test.

Time (day)	0	30	60	90
Content (%)	100.0	101.15	99.98	100.82

**Table 3:** Effect of DQ/HP-*β*-CD/TS ternary complex on *Eimeria tenella* infection (n = 15).

Group	The Total Oocysts (10^8^)	Survival Rate (%)	RWG (%)	CLS	Oocyst Value	ACI
Control	0	100	100	0	0	200
EI	4.51 ± 0.29 ^##^	90	41.97	26.67	40	65.3
DQ	2.70 ± 0.31 **	100	62.85	22.67	20	120.2
DQ/HP-*β*-CD/TS ternary complex	0.51 ± 0.05 **	100	96.48	6.67	5	184.8
Commercial	2.37 ± 0.19 **	100	66.90	21.33	20	125.6

Values are reported as mean ± SEM. ^##^ *p* < 0.01 compared with control group and ** *p* < 0.01 compared with EI group.

**Table 4:** The score of cecal lesions (CLS).

Score	Cecal Characteristics
0	The intestinal contents appear normal, uniform in size, and free of lesions.
1	The intestinal contents appear normal, with a few scattered petechiae on the intestinal wall, and the wall is not thickened.
2	Obvious blood vessels are visible within the intestinal contents, with numerous bleeding points scattered across the intestinal wall, which is slightly thickened and swollen.
3	The intestinal contents include a vast network of blood vessels, some of which appear grayish-white and cheese-like. The intestinal wall is dotted with numerous bleeding points, and the tract itself is congested, swollen, and deformed.
4	The intestine contains a large amount of blood, and even the cecal core and contents are hard, lump-like, and cheese-like. The cecum is obviously atrophied, swollen, and shortened.

## Data Availability

Data is contained in the paper.
